# Outcomes of hyperlactatemia on admission in critically ill patients with acute myocardial infarction: A retrospective study from MIMIC-IV

**DOI:** 10.3389/fendo.2022.1015298

**Published:** 2022-09-23

**Authors:** Ting Lu, Liao Tan, Kai Xu, Jia Liu, Chong Liu, Guogang Zhang, Ruizheng Shi, Zheng Huang

**Affiliations:** ^1^ Department of Cardiology, The Third Xiangya Hospital, Central South University, Changsha, China; ^2^ Department of Critical Care, Changsha Hospital of Traditional Chinese Medicine, Changsha, China; ^3^ Department of Cardiovascular Medicine, Xiangya Hospital, Central South University, Changsha, China; ^4^ Department of Neurosurgery, Xiangya Hospital, Central South University, Changsha, China

**Keywords:** acute myocardial infarction, hyperlactatemia, lactate, mortality, intensive care unit

## Abstract

**Background:**

It has not been verified whether there is a correlation between admission hyperlactatemia and outcomes in critically ill patients with acute myocardial infarction (AMI), especially in large data studies, which we aimed to do in this study.

**Methods:**

For this retrospective study, we extracted analysis data from a famous online intensive care unit database, the Medical Information Mart for Intensive Care (MIMIC)-IV. Included patients were divided into four groups according to the serum lactate level on admission. Hospital mortality and mortality over time were the main outcomes. To explore the relationship between admission hyperlactatemia and outcomes in critically ill patients with AMI, logistic regression, Cox regression, Kaplan-Meier curves, and subgroup analyses were used.

**Results:**

2171 patients matching the selection criteria were enrolled in this study. After adjusting for potential confounding factors, hyperlactatemia on admission contributed to increased short-term mortality in critically ill patients with AMI. The adjusted odds ratio for hospital mortality were 1.62, 3.46 and 5.28 in the mild, moderate, and severe hyperlactatemia groups (95% CI: 1.20-2.18, 2.15-5.58, and 2.20-12.70, respectively). The adjusted hazard ratio for 7-day and 30-day mortality were 1.99 and 1.35 (95% CI: 1.45-2.73 and 1.09-1.67) in the mild hyperlactatemia group, 3.33 and 2.31 (95% CI: 2.22-4.99 and 1.72-3.10) in the moderate hyperlactatemia group, 4.81 and 2.91 (95% CI: 2.86-8.08 and 1.88-4.50) in the severe hyperlactatemia group. The adjusted hazard ratio for 1-year and 5-year mortality were 2.03 and 1.93 (95% CI: 1.58-2.62 and 1.52-2.47) in the moderate hyperlactatemia group, 1.92 and 1.74 (95% CI: 1.28-2.89 and 1.17-2.59) in the severe hyperlactatemia group. Subgroup analyses indicated that the positive correlation between serum lactate level on admission and short-term mortality of critically ill patients with AMI was similar in the subgroups of cardiogenic shock and acute heart failure (*P* for interaction > 0.05).

**Conclusion:**

Hyperlactatemia, especially moderate and severe hyperlactatemia, on admission is closely related to higher short-term mortality incidence in critically ill patients with AMI. The relationship between serum lactate level on admission and short-term mortality of critical AMI patients is stable in subgroups of cardiogenic shock and acute heart failure.

## Introduction

Currently, acute myocardial infarction (AMI) is still a major public problem threatening human life, which kills millions of individuals worldwide every year (1). Although there are huge advancements in medical technology, especially the application of coronary revascularization, the mortality of AMI is still concerning (2). Critically ill patients with AMI have higher mortality incidence with more complications owing to rapid exacerbations and limited time to administer lifesaving treatment (3, 4). Therefore, it is imperative to seek a simple and effective indicator of high mortality incidence in critical AMI patients, to allow early intervention. Increasing evidence has shown that serum biomarkers such as alanine aminotransferase (5), C-reactive protein (6), creatinine clearance (7), and circulating cytochrome C (8) are related to higher mortality incidence in patients with AMI. Unfortunately, the application of these biomarkers is limited owing to time delays or unconventional testing. Hence, a more convenient and effective predictive factor is required to identify “at-risk” patients earlier among critically ill patients with AMI to improve their outcomes.

Lactate, a byproduct of glucose metabolism, is interconverted with pyruvate during anaerobic glycolysis and the Cori cycle (9). Hyperlactatemia, always defined as a lactate level >2 mmol/L (10), occurs when lactate generation exceeds its consumption, and is frequently considered as a biomarker of organ hypoperfusion in many critically ill patients (11). Lactate level >10 mmol/L, described as severe hyperlactatemia, is reportedly significantly associated with high mortality incidence in several critical illnesses (12, 13). With the benefits of being time-saving and easily detectable, serum lactate levels are broadly detected in the intensive care unit (ICU) to evaluate the status of critically ill patients (14). Furthermore, there are increasing reports that elevated lactate level has a significant correlation with cardiovascular disease, including cardiogenic shock post AMI (15), and acute heart failure (16). However, there is still uncertainty whether serum lactate level has a tight correlation with mortality in general AMI patients due to the lack of large-data studies. Therefore, herein, we aim to demonstrate this problem in our study.

## Materials and methods

### Database and study population

The data used in this study were extracted using Medical Information Mart for Intensive Care (MIMIC)-IV v. 2.0. MIMIC-IV is a famous, online accessible clinical critical care database, which is an update version of MIMIC-III containing more than 50000 ICU admissions information for adult patients at the Beth Israel Deaconess Medical Center (Boston, Massachusetts) from 2008 to 2019 (17). To obtain access, author Lu passed the online training courses and exams (certification number: 48803489).

**Figure 1:**
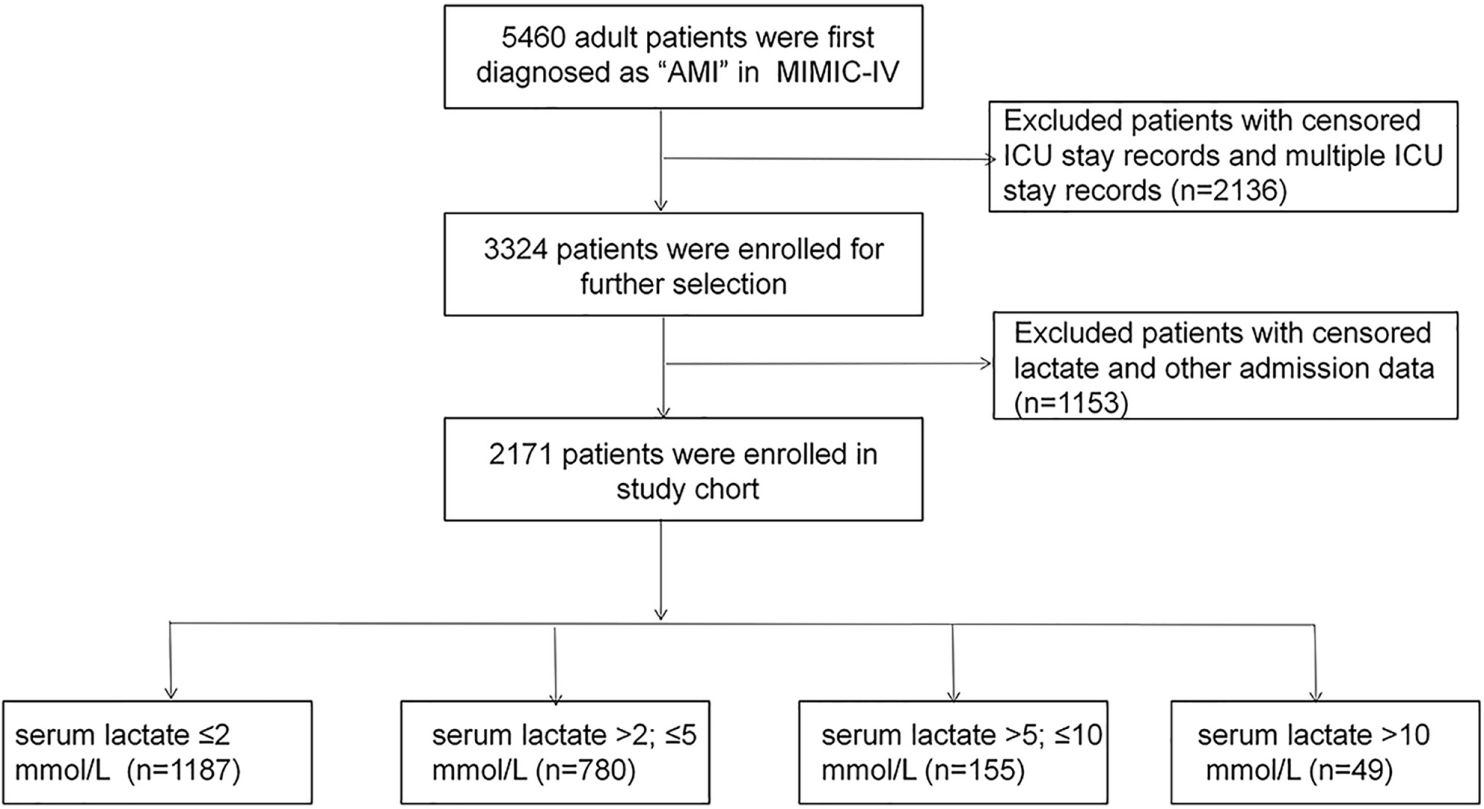
Flowchart of patient selection and grouping.

MIMIC-IV used anonymized personal identifier to protect privacy of all patients. Patients first diagnosed with AMI in MIMIC-IV were selected as the study population. The exclusion criteria were: (1) patients with censored ICU and multiple ICU stay records and (2) patients with censored serum lactate level record on admission and another admission data. A flowchart of patient selection is shown in **Figure 1**.

### Data extraction

PostgreSQL tools (v.14, PostgreSQL Global Development Group, Berkley, California, USA) were used for the data extraction. The diagnosis information of patients was extracted through Structured Query Language (SQL) by International Classification of Disease (ICD) codes, ICD-9 or ICD-10. Laboratory parameters, vital signs indicators, and record of vasoactive drug usage were queried by itemid stored in MIMIC-IV and then matched with the selected patients by ICU stay identity (ID). The disease severity scores, including sequential organ failure assessment (SOFA), systemic inflammatory response (SIRS), and acute physiology score (APS) III, were calculated by scoring laboratory parameters, vital signs and therapy interventions. Query codes were listed in **Supplementary Table 1**. The variables were extracted including: (1) baseline characteristics: age, gender, weight; (2) comorbidities: cardiogenic shock, cardiac arrest, acute heart failure, hypertension, diabetes, prior myocardial infarction, and hypercholesterolemia; (3) vital signs: heart rate, and systolic, diastolic, and mean blood pressures; (4) usage of vasoactive drugs: norepinephrine, dopamine, and epinephrine; (5) records of mechanic ventilation; (6) laboratory parameters: serum lactate, alanine transaminase, white blood cell count (WBC), hemoglobin, creatinine, glucose, arterial oxygen partial pressures (PaO_2_) and bicarbonate; and (7) hospital and ICU stay data: admission and discharge time, ICU in- and out-time, date and time of death. Hospital, 7-day, 30-day, 1-year, and 5-year mortality incidence were arranged as outcome data, which were calculated from hospital stay and follow-up data. Hospital mortality was calculated by hospital expire flag of patients in MIMIC-IV. Mortality incidence at different time was calculated based on the time of first diagnosis of AMI and out-of-hospital date of death in MIMIC-IV 2.0. All measurement parameters used in this study were the first measurements during 24 hours from ICU admission.

Arterial serum lactate level of each patient was collected during 24 hours from ICU admission. In MIMIC-IV, the refer range of lactate was 0.5-2.0 mmol/L. To study the relationship between lactate and mortality in critical AMI patients, referring to definition and grouping of different degrees of hyperlactatemia in previous studies (13, 18–20), the cohort was divided into four groups according to arterial serum lactate level on admission: group I (normal lactate, ≤ 2 mmol/L), group II (mild hyperlactatemia, >2 and ≤ 5 mmol/L), group III (moderate hyperlactatemia, >5 and ≤ 10 mmol/L), and group IV (severe hyperlactatemia, >10 mmol/L).

### Statistical analysis

Continuous variables in baseline characteristic were non-normal distribution, which were presented as median with interquartile range. Kruskal-Wallis test was used in group comparison. Categorical variables were presented as counts and percentages, which compared by Pearson’s chi-squared test. Logistic regression analyses with results expressed as odds ratios (OR) with 95% confidence intervals (CI) and Cox proportional hazards regression analyses with results expressed as hazard ratios (HR) with 95% CI were used to assess the relationship between serum lactate level and mortality. Univariate analyses were used to explore the variables associated with death (*P* < 0.05). Statistically significant variables were brought into multivariate analysis as covariates for further analysis. The cumulative risk of death within the four lactate groups were presented using Kaplan-Meier curves. Subgroup analyses were used to assess whether serum lactate level was associated with mortality in subgroups of cardiogenic shock and acute heart failure, which was analyzed by multivariate regression analysis. Statistical analyses were performed using the Stata software (ver.16.0, Stata Corp, Texas, American). Figures were performed using R (v 4.2.1, The R Foundation, Auckland, New Zealand). Two-tailed *P* < 0.05 was statistically significant.

## Results

### Population characteristics

We extracted data of 2171 eligible patients from the MIMIC-IV (**Figure 1**). Based on the serum lactate level on admission, the patients were categorized into four groups: 1187 patients in group I (lactate ≤ 2 mmol/L), 780 patients in group II (lactate >2 and ≤ 5 mmol/L), 155 patients in group III (lactate >5 and ≤10 mmol/L), and 49 patients in group IV (lactate >10 mmol/L). **Table 1** presented the baseline characteristics of the included population. The median age of the included patients were 69 years old (interquartile range, 59–77). Comparison between groups showed no statistically significant differences in the age, sex, or comorbidities of hypertension, diabetes, hypercholesterolemia, and prior myocardial infarction (all *P* > 0.05). The incidence of comorbidities of acute cardiac events, including cardiogenic shock, cardiac arrest, and acute heart failure, increased with the severity of hyperlactatemia (all *P* < 0.05). The usage of vasoactive drugs, including norepinephrine, dopamine, and epinephrine, also increased with the severity of hyperlactatemia (all *P* < 0.001). APS III and SOFA are classic critical scoring tools which are widely used for assessment of severity of critically ill patients, including AMI patients (21–23). Patients in the more severe hyperlactatemia groups got higher severity scores, including APS III, SIRS, and SOFA scores (all *P* < 0.001). Moreover, with increasing severity of hyperlactatemia, patients exhibited more unstable vital signs, hepatic and renal dysfunction, higher white cell counts, lower PaO_2_, and higher glucose levels (all *P* < 0.001).

**Table 1:** Baseline characteristics and outcomes of critically ill patients with AMI according to serum lactate level on admission in MIMIC-IV database.

Variables	All patients(n=2171)	serum lactate level (mmol/L)				*P*
		≤2 (n = 1187)	>2; ≤5 (n = 780)	>5; ≤10 (n = 155)	>10 (n = 49)	
Age (years)	69 (60-78)	69 (59-77)	69 (60-79)	70 (62-80)	70 (61-79)	0.482
Weight (kg)	79.7 (67.9-94.0)	80.0 (68.9-96.3)	78.7 (66.0-91.6)	78.0 (66.8-90.4)	79.8 (68.9-94.8)	0.009
Gender, n (%)						0.252
Male	1426 (65.68%)	799 (67.31%)	499 (63.97%)	100 (64.52%)	28 (57.14%)	
Female	744 (34.27%)	388 (32.69%)	281 (36.03%)	55(35.48%)	21 (42.86%)	
Comorbidities, n (%)						
Cardiogenic shock	474 (21.83%)	187 (15.75%)	179 (22.95%)	80 (51.61%)	28 (57.24%)	<0.001
Acute heart failure	762 (35.10%)	445 (37.49%)	263 (33.72%)	43 (27.74%)	11 (22.45%)	0.013
Cardiac arrest	167 (7.69%)	59 (4.97%)	68 (8.72%)	31 (20.00%)	9 (18.34%)	<0.001
Diabetes	775 (35.75%)	429 (36.14%)	279 (35.77%)	45 (29.03%)	22 (44.90%)	0.178
Hypertension	768 (35.38%)	419 (35.30%)	288 (36.92%)	52 (33.55%)	9 (18.37%)	0.064
Hypercholesterolemia	102 (4.70%)	60 (5.05%)	33 (4.23%)	6 (3.87%)	3 (6.12%)	0.759
Prior MI	297 (13.69%)	165 (13.90%)	110 (14.10%)	16 (10.32%)	6 (12.24%)	0.630
Vital signs						
Heart rate (bpm)	85 (75-98)	82 (73-95)	85 (76-99)	93 (81-112)	97 (78-110)	<0.001
SBP (mmHg)	116 (102-132)	118 (104-133)	114 (101-130)	111 (98-127)	102 (88-121)	<0.001
DBP (mmHg)	63 (53-76)	64 (54-76)	63 (53-75)	66 (52-78)	58 (51-66)	0.045
MBP (mmHg)	81 (70-91)	81 (71-92)	80 (69-90.5)	80 (67-89)	73 (63-82)	0.003
Use of vasoactive drugs (n (%))						
Norepinephrine	846 (38.97%)	350 (29.49%)	340 (43.59%)	113 (72.90%)	43 (87.76%)	<0.001
Dopamine	238 (10.96%)	89 (7.50%)	98 (12.56%)	37 (23.87%)	14 (28.57%)	<0.001
Epinephrine	328 (15.11%)	137 (11.54%)	122 (15.64%)	44 (28.39%)	25 (51.02%)	<0.001
Laboratory parameters						
Lactate (mmol/L)	1.9 (1.4-3.0)	1.4 (1.1-1.7)	2.8 (2.4-3.5)	6.9 (5.8-8.2)	13.2 (11.2-15.5)	<0.001
WBC (K/µL)	12.2 (8.9-16.6)	11.3 (8.5-15.1)	12.9 (9.2-17.7)	14.0 (10.2-20.0)	17.4 (12.7-23.0)	<0.001
Hemoglobin (g/dL)	10.8 (8.9-12.8)	10.7 (8.9-12.7)	10.8 (8.9-13.0)	11.5 (9.5-13.2)	9.9 (8.0-11.9)	0.018
ALT (IU/L)	55 (22-99)	44 (20-58)	63 (23-153)	166 (39-602)	587 (93-1255)	<0.001
Creatinine (mg/dL)	1.2 (0.9-1.8)	1.1 (0.9-1.7)	1.2 (0.9-1.7)	1.6 (1.2-2.3)	1.9 (1.5-3.0)	<0.001
Glucose (mg/dL)	143 (114-201)	133 (109-174)	154.5 (118-224)	209 (147-307)	243 (153-400)	<0.001
PaO_2_ (mmHg)	92 (71-104)	99 (74-108)	91 (70-104)	76 (60-86)	69 (55-84)	<0.001
Bicarbonate (mmol/L)	22 (19-24)	22 (20-25)	21 (19-24)	17 (14-21)	12 (10-16)	<0.001
Severity Score						
APS III	49 (34-71)	44 (32-60)	51 (36-76)	84 (56-102)	93 (76-106)	<0.001
SIRS	3 (2-3)	3 (2-3)	3 (2-3)	3 (3-4)	3 (3-4)	<0.001
SOFA	6 (4-9)	5 (3-8)	7 (4-10)	12 (7-14)	13 (10-15)	<0.001
Outcomes						
Hospital mortality (n (%))	449 (20.75%)	133 (11.20%)	184 (23.59%)	94 (60.65%)	38 (77.55%)	<0.001
Hospital LOS (day)	8.7 (5.2-13.1)	9.0 (5.9-13.7)	8.6 (5.1-12.9)	5.2 (1.4-13.8)	1.2 (0.4-9.3)	<0.001
7-day mortality (n (%))	299 (13.77%)	66 (5.56%)	121 (15.51%)	77 (49.68%)	35 (71.43%)	<0.001
30-day mortality (n (%))	523 (24.09%)	179 (15.08%)	203 (26.03%)	103 (66.45%)	38 (77.55%)	<0.001
1-year mortality (n (%))	755 (34.78%)	320 (26.96%)	278 (35.64%)	119 (76.77%)	38 (77.55%)	<0.001
5-year mortality (n (%))	852 (39.24%)	383 (32.27%)	308 (39.49%)	122 (78.71%)	39 (79.59%)	<0.001

MI, myocardial infarction; SBP, systolic blood pressure; DBP, diastolic blood pressure; MBP, mean blood pressure; WBC, white blood cell; ALT, alanine transaminase; PaO_2_, arterial oxygen partial pressures; APS III, Acute Physiology Score; SIRS, systemic inflammatory response; SOFA, sequential organ failure assessment; LOS, length of stay.

### Association between hyperlactatemia on admission and clinical outcomes in critically ill patients with AMI

**Figure 2:**
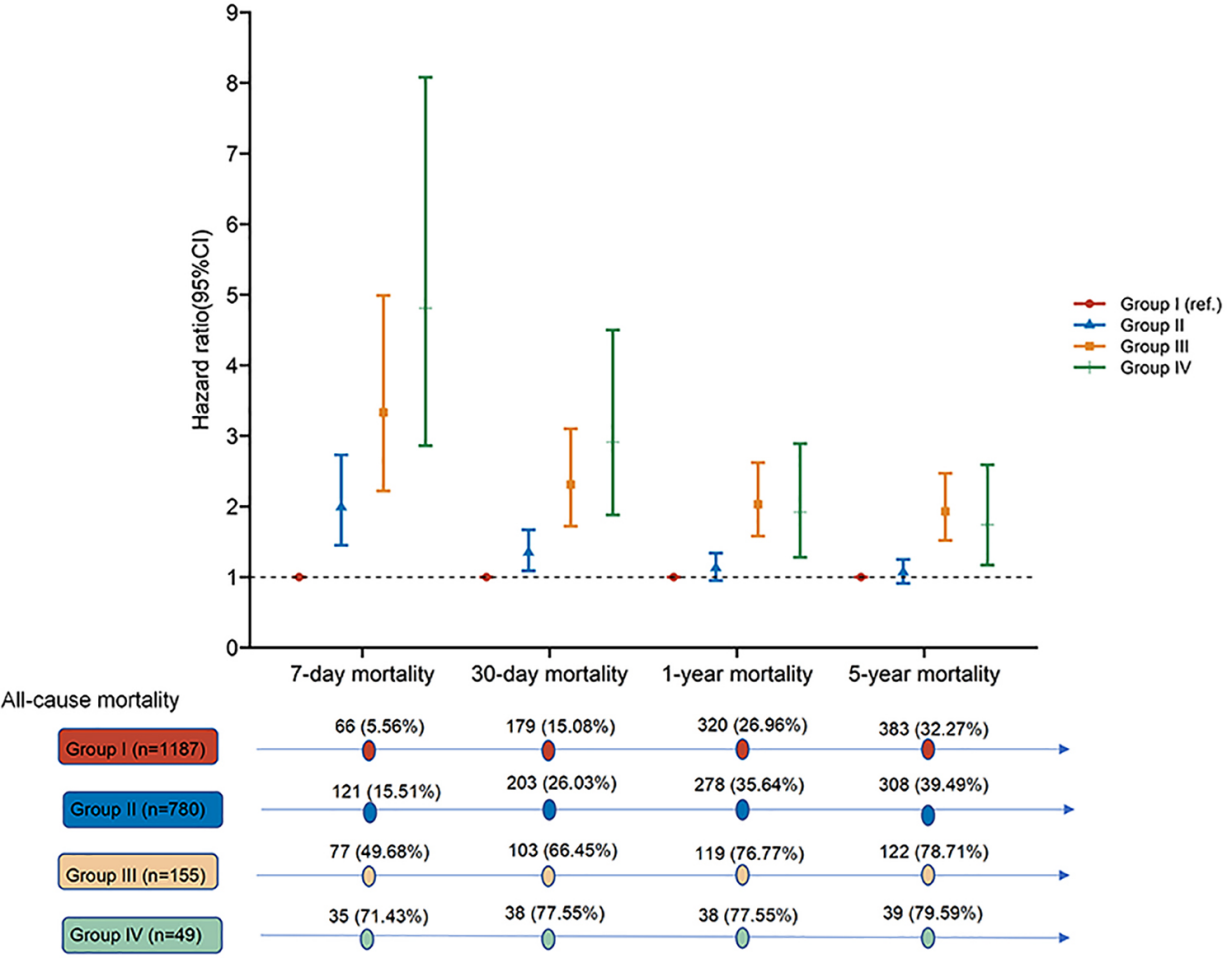
Hazard ratio for all-cause mortality.

We found that mortality incidence of critical AMI patients was still high; 20.75% of the enrolled patients died in the hospital, and 39.24% of the patients died within 5 years after primary hospital admission. After comparing the crude mortalities of the different groups, we found that patients in groups II, III, and IV (hyperlactatemia) presented a higher risk of death than those in group I (normal lactate level) (**Table 1** and **Figure 2**). The short- and long-term mortality incidence showed a significant upward trend as the severity of hyperlactatemia increased, especially in groups III and IV (7-day mortality: 49.68% vs. 71.43%; 30-day mortality: 66.45% vs. 77.55%; 1-year mortality: 76.77% vs. 77.55%; 5-year mortality: 78.71% vs. 79.59%).

The association between admission serum lactate level and hospital mortality was identified by logistic regression analysis (**Table 2**). In the univariate logistic regression analysis, groups II, III, and IV showed a high predictive power for hospital mortality (OR, 95% CI: 2.45, 1.92-3.12; 11.21, 8.44-17.67; 27.38, 13.67-54.85, respectively). In the multivariate logistic regression analysis, after adjusting for the variables related to death which were statistically significant in prior univariable logistic regression analysis for hospital mortality (list in **Supplementary Table 2**, *P* < 0.05), including age, sex, cardiogenic shock, cardiac arrest, diabetes, hypertension, heart rate, systolic and mean blood pressure, vasoactive drug usage, ALT, white cell count, creatinine, glucose, PaO_2_, bicarbonate, SIRS, APS III, and SOFA, the adjusted OR value decreased, but it still performed high predictive validity of hospital mortality in patients in groups III and IV (OR, 95% CI: 3.46, 2.15-35.58; 5.28, 2.20-12.70, respectively).

**Table 2:** Univariable and multivariable logistic regression analyses for hospital mortality of critically ill patients with AMI according to serum lactate level on admission.

Groups	Univariable Model			Multivariable Model		
	ORs	95%CI	*P*	ORs	95%CI	*P*
Group I	1.0(ref)	/	/	1.0(ref)	/	/
Group II	2.45	1.92-3.12	<0.001	1.62	1.20-2.18	0.002
Group III	11.21	8.44-17.67	<0.001	3.46	2.15-5.58	<0.001
Group IV	27.38	13.67-54.85	<0.001	5.28	2.20-12.70	0.005

Group I: Admission serum lactate ≤2mmol/L; Group II: Admission serum lactate >2; ≤5mmol/L; Group III: Admission serum lactate >5; ≤10mmol/L; Group IV: Admission serum lactate >10mmol/L.

Adjusted variables for the multivariate regression analysis: age, sex, cardiogenic shock, cardiac arrest, diabetes, hypertension, heart rate, systolic and mean blood pressure, vasoactive drug usage, ALT, white cell count, creatinine, glucose, PaO_2_, bicarbonate, SIRS, APS III, and SOFA.

**Figure 3:**
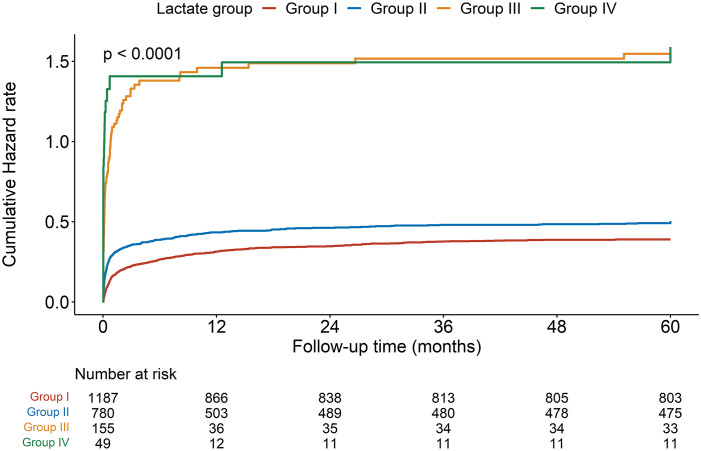
Kaplan-Meier curve for cumulative hazard of 5-year mortality.

Kaplan-Meier curves presented significantly higher cumulative risk of death over 5 years in groups III and IV (**Figure 3**). In the univariable Cox regression analysis, the risk of short-term mortality of patients in groups II, III, and IV was much higher (HR, 95% CI; 7-day mortality: 2.95, 2.19-3.99; 12.01, 8.64-16.70; 22.72, 15.03-34.35; 30-day mortality: 1.89, 1.55-2.31; 7.12, 5.58-9.08; 11.55, 8.12-16.44, respectively). There was a similar trend in the 1-year mortality in groups II, III, and IV (HR, 95% CI: 1.47, 1.25-1.72; 5.37, 4.35-6.64; 7.15, 5.10-10.02, respectively). In the 5-year mortality analysis, unlike groups III and IV (HRs, 95% CI: 4.83, 3.93-5.93 vs 6.27, 4.50-8.73, respectively), admission lactate level in group II lost risk prediction power (*P* = 0.485).

In the multivariable Cox regression analysis (**Table 3** and **Figure 2**), after adjusting for the variables related to death which were statistically significant in prior univariable Cox regression analysis for mortalities (**Supplementary Table 3**), including age, sex, weight, comorbidities, heart rate, systolic and mean blood pressure, vasoactive drug usage, ALT, white cell count, creatinine, glucose, PaO_2_, bicarbonate, SIRS, APS III, and SOFA, short-term mortality incidence was still high for patients in groups II, III and IV; the risk increased with hyperlactatemia severity (HR, 95% CI; 7-day mortality: 1.99, 1.45-2.73; 3.33, 2.22-4.99; 4.81, 2.86-8.08; 30-day mortality: 1.35, 1.09-1.67; 2.31, 1.72-3.10; 2.91, 1.88-4.50, respectively). In the long-term mortality analysis, the predictive power varied among the different groups. The adjusted HR for 1-year mortality was 2.03 (95% CI: 1.58-2.62) in group III and 1.92 (95% CI: 1.28-2.89) in group IV, but did not have predict power in group II (*P* = 0.158). Analysis of the 5-year mortality revealed a similar trend. The adjusted HR of group III was 1.93 (95% CI: 1.52-2.47), and of group IV was 1.74 (95% CI: 1.17-2.59). However, predict power was lost in group II (*P* = 0.419).

**Table 3:** Univariable and multivariable Cox regression analyses for outcomes of critically ill patients with AMI according to serum lactate level on admission.

Outcomes	Univariable Model			Multivariable Model			
groups	HR	95%CI	*P*	HR	95%CI	*P*
7-day mortality						
Group I	1.0 (ref)	/	/	1.0 (ref)	/	/
Group II	2.95	2.19-3.99	<0.001	1.99	1.45-2.73	<0.001
Group III	12.01	8.64-16.70	<0.001	3.33	2.22-4.99	<0.001
Group IV	22.72	15.03-34.35	<0.001	4.81	2.86-8.08	<0.001
30-day mortality						
Group I	1.0 (ref)	/	/	1.0 (ref)	/	/
Group II	1.89	1.55-2.31	<0.001	1.35	1.09-1.67	0.007
Group III	7.12	5.58-9.08	<0.001	2.31	1.72-3.10	<0.001
Group IV	11.55	8.12-16.44	<0.001	2.91	1.88-4.50	<0.001
1-year mortality						
Group I	1.0 (ref)	/	/	1.0 (ref)	/	/
Group II	1.47	1.25-1.72	<0.001	1.13	0.95-1.34	0.158
Group III	5.37	4.35-6.64	<0.001	2.03	1.58-2.62	<0.001
Group IV	7.15	5.10-10.02	<0.001	1.92	1.28-2.89	0.002
5-year mortality						
Group I	1.0 (ref)	/	/	1.0 (ref)	/	/
Group II	1.36	1.17-1.58	0.485	1.07	0.91-1.25	0.419
Group III	4.83	3.93-5.93	<0.001	1.93	1.52-2.47	<0.001
Group IV	6.27	4.50-8.73	<0.001	1.74	1.17-2.59	0.006

Group I: Admission serum lactate ≤2mmol/L; Group II: Admission serum lactate >2; ≤5mmol/L; Group III: Admission serum lactate >5; ≤10mmol/L; Group IV: Admission serum lactate >10mmol/L.

Adjusted variables for the multivariate regression analyses: age, sex, weight, cardiogenic shock, cardiac arrest, acute heart failure (only in 7-day mortality and 5-year mortality analysis), diabetes (only in 7-day mortality and 30-day mortality analysis), hypertension, vasoactive drug usage, ALT, white cell count, creatinine, glucose, PaO_2_, bicarbonate, hemoglobin (only in 5-year mortality analysis), heart rate, systolic blood pressure, mean blood pressure (except 1-year mortality analysis), SIRS, APS III, and SOFA.

### Subgroup analyses of the association between serum lactate level on admission and mortalities

**Figure 4:**
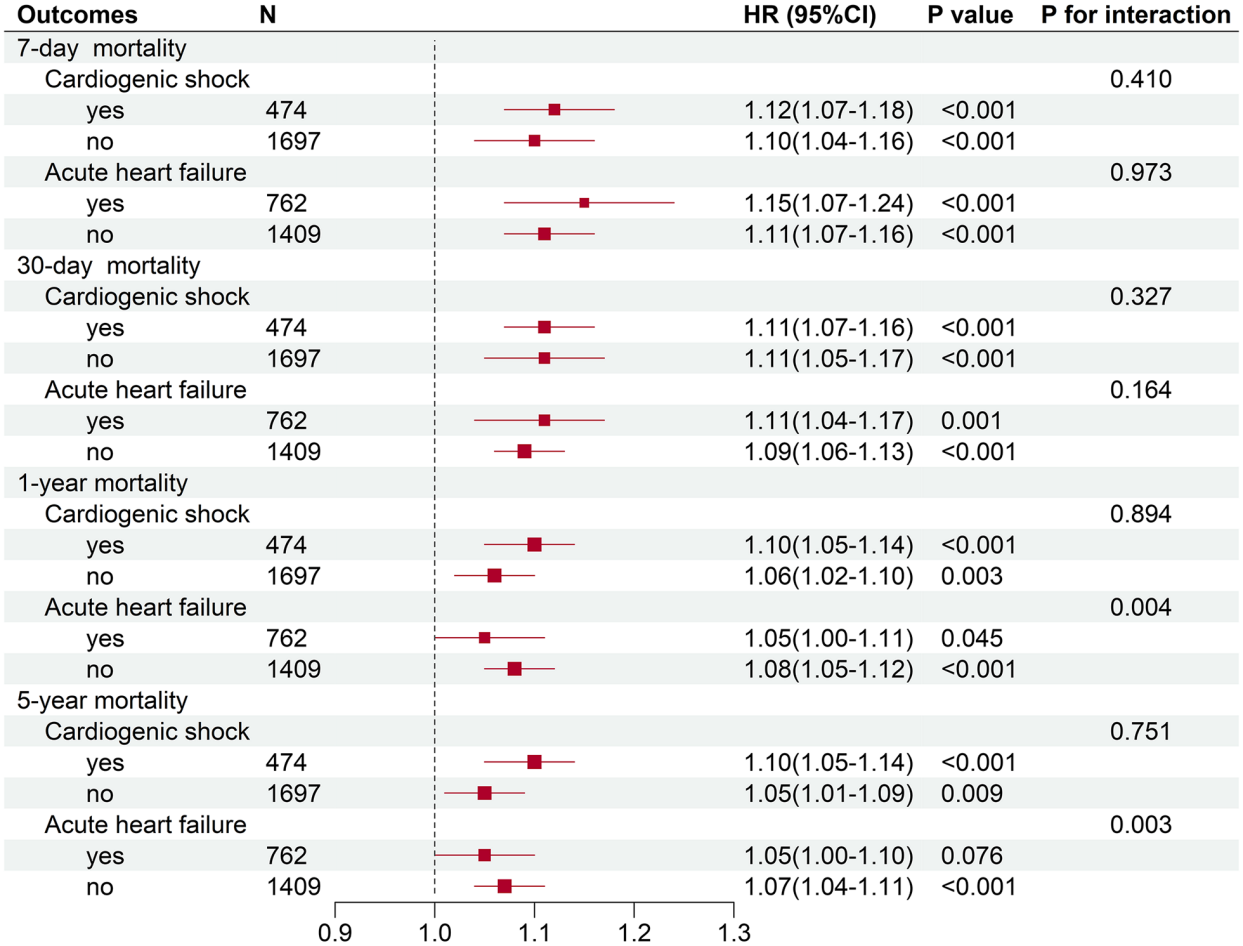
Subgroup analyses of association between serum lactate level on admission and different time mortalities in critically ill patients with AMI.

To further clarify whether the association between serum lactate level on admission and mortality in critical AMI patients was affected by acute cardiac comorbidities, subgroup analyses were conducted. It had been observed that the association between admission serum lactate level and different time mortalities had not been affected by incidence of cardiogenic shock (*P* for interaction: 0.278-0.894) (**Table 4** and **Figure 4**). In the acute heart failure subgroup, there were no statistically significant differences in hospital, 7-day, and 30-day mortality incidence (*P* for interaction: 0.065-0.973). However, the relationship between serum lactate level on admission and the 1-year and 5-year mortality analysis showed statistically significant differences in acute heart failure subgroup (*P* for interaction < 0.05). In the 1-year and 5-year mortality prediction, admission lactate was only correlated with mortality in critical AMI patients without acute heart failure (*P* < 0.001).

**Table 4:** Subgroup analysis of association between serum lactate level on admission and hospital mortality in critically ill patients with AMI.

	n	OR	95%CI	*P*	*P* for interaction
Cardiogenic shock					0.287
NO	1697	1.22	1.12-1.34	<0.001	
YES	474	1.19	1.08-1.31	0.001	
Acute heart failure					0.065
NO	1409	1.23	1.13-1.34	<0.001	
YES	762	1.14	1.02-1.28	0.020	

## Discussion

Our study from MIMIC-IV data demonstrated that mortality incidence of critical AMI patients was still high, with over 20% death in hospital and the incidence approaching 40% in five years. Hyperlactatemia on admission was associated with short-term mortality in critical AMI patients, and those with moderate and severe hyperlactatemia had a higher risk of death. Through subgroup analysis, we proved that the relationship between serum lactate level on admission and short-term mortality was no differences in cardiogenic shock and acute heart failure groups of critical AMI patients. To our knowledge, this study is the first to report the association between serum lactate level on admission and different time mortalities in critical AMI patients using a large ICU database.

Serum lactate, a sign of microcirculatory hypoperfusion, is widely used in ICU to evaluate the severity of patients and provide guidance for circulatory resuscitation, especially in patients with sepsis (24). Serum lactate is a facile indicator, but is not commonly used in evaluation of critical AMI patients. We extracted 3324 patients from the MIMIC-IV database who were first diagnosed of AMI and with an ICU admission; only 2171 patients had a recorded lactate level within 24 hours after ICU admission. Current researches on the relationship between lactate and the prognosis of critical AMI patients primarily focused on patients complicated with cardiogenic shock or acute heart failure. In patients with cardiogenic shock, several studies indicated elevated admission lactate level was a strong predictor of 30-day mortality (25, 26). In a Swedish study, lactate ≥ 2.5 mmol/L was significantly associated with 30-day mortality in patients with AMI (Killip class II-III) (27). However, the relationship was not well studied in the general AMI population. We have to realize that there are numerous patients with AMI without any cardiac complications whose lactate levels are increased as well (28–30). In our study, we found that hyperlactatemia on admission was associated with mortality in the general population with AMI. In the subgroup analyses, there was no significant differences between admission lactate level and short- and long-term mortality incidence in the cardiogenic shock subgroups, suggesting that lactate level on admission was a reliable predictor of mortality in critical AMI patients, regardless of cardiogenic shock. We noted differences in the short- and long-term mortality incidence between patients with critical AMI in the acute heart failure subgroup. The long-term prognosis of acute heart failure, post-AMI, is affected by multiple risk factors and drug intervention (31), which might weaken the predictive power of lactate level on admission.

Previous studies on the relationship between lactate levels and mortality due to cardiovascular disease had mainly focused on short-term mortality, especially the 30-day mortality. In a single-center, retrospective, observational study from China, elevated admission lactate level was reportedly associated with 30-day mortality of acute coronary syndrome patients in Coronary Care Unit (32). Frydland et al. determined that admission serum lactate level >2 mmol/L was a valuable predictor of 30-day mortality in patients with AMI (28). In our study, we found that patients with hyperlactatemia on admission had a high risk of death within 30 days after hospitalization, especially in those with moderate and severe hyperlactatemia. Hyperlactatemia on admission was also strongly associated with hospital mortality in critical AMI patients. Considering that the median length of hospital stay in the enrolled patients was 8.7 days, we believe that moderate and severe hyperlactatemia on admission may be a strong predictor of short-term mortality. Studies of the relationship between lactate levels and long-term mortality in critically ill patients were much less. A German study reported that delta-lactate with 24-hour of admission was associated with higher one-year mortality incidence in critically ill patients (11). In a single-center study of patients with acute heart failure, those with elevated lactate levels on admission were reported to have a 2.7-fold increase in 1-year mortality incidence (16). Studies on the relationship between lactate levels and 5-year mortality in patients with AMI were rare. In the results of our study, we found that 383 of 1187 patients with mild hyperlactatemia died within five years after primary admission, but HR of mild hyperlactatemia was not statistically significant in the 1- and 5-year mortality prediction. Moderate and severe hyperlactatemia remained predictors of the 5-year mortality in our study. Considering that more than 60% of patients with moderate and severe hyperlactatemia died within 30 days, the association between moderate and severe hyperlactatemia and long-term mortality in critical AMI patients needs to be further verified in larger populations.

Lactate is a product of glycolysis, whose increased concentration reflects the imbalance in lactate production and metabolism (11). High serum lactate results from tissue hypoperfusion and hypoxia due to circulatory failure (33). In cardiogenic shock, the decrease of cardiac output and organ perfusion due to poor systolic function increases serum lactate levels (14). However, further studies show that plasma lactate levels elevate not only in tissue hypoperfusion, but also in situations of stress (34). Serum catecholamines are increased in patients with AMI (35) and in AMI animal models (36). Elevated epinephrine levels activate β2 adrenergic receptors, thereby activating glycolytic metabolism, and promoting lactate production (9). Studies have shown a positive linear correlation between lactate levels and epinephrine levels in the body during exercise (37). Ruptured coronary plaques release inflammatory factors into the blood circulation to induce SIRS in patients with AMI (38). SIRS can lead to hemodynamic instability (38) and liver dysfunction (39), thereby resulting in hyperlactatemia due to increasing plasma lactate production and decreasing clearance. In our study, patients with moderate and severe hyperlactatemia had higher SIRS scores and elevated liver transaminases, which may further support the role of inflammation and liver dysfunction in hyperlactatemia. It can be concluded that, the elevated serum catecholamine levels and an enhanced inflammatory response post myocardial infarction might be the main reasons for the elevated serum lactate levels in AMI patients.

There are some limitations that should be noted in our study. First, this is a retrospective, observational study with inevitable selection bias. Second, due to missing lactate values, our study mainly focused on the relationship between serum lactate level on admission and mortality in critical AMI patients. Though, it has been reported that lactate clearance (40, 41) also has a good performance in prognostic prediction in critically ill patients. The relationship between lactate levels and prognosis of patients with AMI needs to be further validated by prospective cohort studies in the future.

## Conclusion

Hyperlactatemia, especially moderate and severe hyperlactatemia, on admission is closely related to higher short-term mortality incidence in critically ill patients with AMI. The relationship between serum lactate level on admission and short-term mortality of critical AMI patients is stable in subgroups of cardiogenic shock and acute heart failure.

## Data availability statement

The original contributions presented in the study are included in the article/**Supplementary Material**. Further inquiries can be directed to the corresponding authors.

## Ethics statement

MIMIC IV is a publicly, available ICU database with anonymous patient information, approved ethical review and data sharing. Written informed consent for participation was not required for this study in accordance with the national legislation and the institutional requirements.

## Author contributions

TL contributed to the research design, data extraction, data analysis, paper writing; LT contributed to the research design, data extraction, and data analysis; KX, JL and CL contributed to paper writing; GZ, RS, and ZH contributed to administrative support and revision of the manuscript. All authors read and agreed to publish the final manuscript.

## Funding

The work was supported by the National Nature Science Foundation of Changsha (Nos. Kq2014254).

## Conflict of interest

The authors declare that the research was conducted in the absence of any commercial or financial relationships that could be construed as a potential conflict of interest.

## Publisher’s note

All claims expressed in this article are solely those of the authors and do not necessarily represent those of their affiliated organizations, or those of the publisher, the editors and the reviewers. Any product that may be evaluated in this article, or claim that may be made by its manufacturer, is not guaranteed or endorsed by the publisher.

## Abbreviations

MIMIC, Medical Information Mart for Intensive Care; ICU, intensive care unit; ICD, International Classification of Disease; AMI, acute myocardial infarction; ALT, alanine aminotransferase; OR, odds ratio; CI, confidence interval; HR, hazards ratio; MI: myocardial infarction; SBP, systolic blood pressure; DBP, diastolic blood pressure; MBP, mean blood pressure; WBC, white blood cell; ALT, alanine transaminase; PaO_2_, arterial oxygen partial pressures; APS III, Acute Physiology Score; SIRS, systemic inflammatory response; SOFA, sequential organ failure assessment.
