# Supplementary material for: Outcomes of hyperlactatemia on admission in critically ill patients with acute myocardial infarction: A retrospective study from MIMIC-IV
Source: Front Endocrinol (Lausanne). 2022 Sep 23;13:1015298. doi: 10.3389/fendo.2022.1015298 (PMC9538672; doi:10.3389/fendo.2022.1015298)
Supplement: Supplementary file 1 [file DataSheet_1.docx]

Supplementary Material

# Supplementary Tables：

**Supplementary Table 1. Variables query code in MIMIC-IV**

| Variables | Query code |
| --- | --- |
| Acute myocardial infarction | 41001, 41002, 41011, 41012, 41021, 41022, 41031, 41041, 41042, 41051, 41081, 41082, 41091, 41092, I2101, I2102, I2109, I2111, I2119, I2121, I2129, I213, I214, I219, I21A1, I21A9, I222 |
| Hypertension | 4010, 4011, 4019, 40591, I10 |
| Cardiogenic shock | 78551, R570 |
| Cardiac arrest | 4275, I46 |
| Acute heart failure | 42821, 42823, 42831, 42833, 42841, 42843, I5021, I5023, I5031, I5033, I5043 |
| Diabetes | E10, E11, E13 |
| Prior myocardial infarction | 412, I252 |
| Hypercholesterolemia | 2720, E780, E7800 |
| norepinephrine | 221906 |
| dopamine | 221662 |
| epinephrine | 221289 |
| Temperature | 223761, 23762 |
| Weight (Kg) | 226512 |
| GCS | 220739, 223900, 223901 |
| Respirate rate | 220210 |
| Urine output | 226560 |
| hematocrit | 51221 |
| platelet | 51265 |
| bilirubin | 50885 |
| ventilation | 225792 |
| Oxygen | 50816 |
| Lactate (mmol/L) | 50813 |
| WBC (K/µL) | 51755 |
| Hemoglobin (g/dL) | 220228 |
| ALT (IU/L) | 50861 |
| Creatinine (mg/dL) | 52024 |
| Glucose (mg/dL) | 220621 |
| PaO_2_ (mmHg) | 50821 |
| Bicarbonate (mmol/L) | 50803 |
| Urea nitrogen (mg/dl) | 51006 |
| Sodium (mEq/L) | 50983 |
| Albumin (g/dL) | 50862 |
| Heart rate (bpm) | 220045 |
| SBP (mmHg) | 220050 |
| DBP (mmHg) | 220051 |
| MBP (mmHg) | 220052 |

GCS: Glasgow Coma Scale; SBP: systolic blood pressure, DBP: diastolic blood pressure, MBP: mean blood pressure, WBC: white blood cell; ALT: alanine transaminase*;* PaO_2_: arterial oxygen partial pressures;

**Supplementary Table 2. Univariable Logistic regression analyses for hospital mortality in critically ill patients with AMI**

| Variable | ORs | 95%CI | *P* |
| --- | --- | --- | --- |
| Age (years) | 1.03 | 1.02-1.04 | <0.001 |
| Weight (kg) | 1.00 | 0.99-1.00 | 0.157 |
| Gender | 1.44 | 1.16-1.78 | 0.001 |
| Cardiogenic shock | 4.13 | 3.30-5.18 | <0.001 |
| Acute heart failure | 0.94 | 0.76-1.18 | 0.610 |
| Cardiac arrest | 4.93 | 3.57-6.82 | <0.001 |
| Diabetes | 0.65 | 0.52-0.82 | <0.001 |
| Hypertension | 0.62 | 0.49-0.78 | <0.001 |
| Hypercholesterolemia | 0.76 | 0.45-1.29 | 0.307 |
| Prior MI | 1.04 | 0.77-1.40 | 0.808 |
| Norepinephrine | 6.57 | 5.20-8.31 | <0.001 |
| Dopamine | 4.39 | 3.31-5.80 | <0.001 |
| Epinephrine | 2.40 | 1.86-3.10 | <0.001 |
| SOFA | 1.30 | 1.27-1.34 | <0.001 |
| SIRS | 1.64 | 1.45-1.87 | <0.001 |
| APS III | 1.05 | 1.04-1.05 | <0.001 |
| WBC (K/µL) | 1.03 | 1.01-1.04 | <0.001 |
| Hemoglobin (g/dL) | 1.02 | 0.98-1.06 | 0.364 |
| Glucose (mg/dL) | 1.00 | 1.00-1.00 | <0.001 |
| Creatinine (mg/dL) | 1.15 | 1.09-1.21 | <0.001 |
| PaO_2_ (mmHg) | 0.99 | 0.99-1.0 | <0.001 |
| Bicarbonate (mmol/L) | 0.88 | 0.86-0.90 | <0.001 |
| ALT (IU/L) | 1.00 | 1.00-1.00 | <0.001 |
| Heart rate (bpm) | 1.02 | 1.01-1.02 | <0.001 |
| SBP (mmHg) | 0.99 | 0.99-1.00 | <0.001 |
| DBP (mmHg) | 1.00 | 0.99-1.00 | 0.511 |
| MBP (mmHg) | 0.99 | 0.99-1.00 | 0.004 |

MI: myocardial infarction; SOFA: sequential organ failure assessment; SIRS: systemic inflammatory response; APS III: Acute Physiology Score; WBC: white blood cell; PaO_2_: arterial oxygen partial pressures; ALT: alanine transaminase; SBP: systolic blood pressure, DBP: diastolic blood pressure, MBP: mean blood pressure,

**Supplementary Table 3. Univariable Cox regression analyses for outcomes in critically ill patients with AMI**

| Variable | 7-day mortality  HR (95%CI), *P* | 30-day mortality HR (95%CI), *P* | 1-year mortality HR (95%CI), *P* | 5-year mortality HR (95%CI), *P* |
| --- | --- | --- | --- | --- |
| Age (years) | 1.03 (1.02-1.04), <0.001 | 1.03 (1.02-1.04), <0.001 | 1.03 (1.03-1.04), <0.001 | 1.03 (1.03-1.04), <0.001 |
| Weight (kg) | 0.99 (0.99-1.00), 0.003 | 0.99 (0.99-1.00), 0.002 | 0.99 (0.99-0.99), <0.001 | 0.99 (0.99-0.99), <0.001 |
| Gender | 1.68 (1.34-2.11), <0.001 | 1.38 (1.16-1.64), <0.001 | 1.48 (1.28-1.71), <0.001 | 1.50 (1.31-1.72), <0.001 |
| Cardiogenic shock | 3.70 (2.95-4.64), <0.001 | 3.38 (2.84-4.01), <0.001 | 2.64 (2.27-3.06), <0.001 | 2.49 (2.16-2.87), <0.001 |
| Acute heart failure | 0.66 (0.51-0.86), 0.002 | 1.01 (0.85-1.21), 0.899 | 1.21(1.05-1.40), 0.009 | 1.28 (1.11-1.47), <0.001 |
| Cardiac arrest | 3.65 (2.76-4.83), <0.001 | 3.26 (2.59-4.01), <0.001 | 2.89 (2.36-3.55), <0.001 | 2.70 (2.21-3.29), <0.001 |
| Diabetes | 0.67 (0.52-0.86), 0.002 | 0.73 (0.61-0.86), 0.001 | 0.89 (0.76-1.03), 0.125 | 0.92 (0.80-1.06), 0.125 |
| Hypertension | 0.66 (0.51-0.85), 0.002 | 0.62 (0.51-0.75), <0.001 | 0.58 (0.49-0.68), <0.001 | 0.57 (0.49-0.67), <0.001 |
| Hypercholesterolemia | 0.69 (0.37-1.30), 0.255 | 0.93 (0.61-1.41), 0.720 | 1.11 (0.80-1.53), 0.537 | 1.20 (0.89-1.61), 0.225 |
| Prior MI | 1.08 (0.79-1.48), 0.631 | 1.00 (0.78-1.29), 0.975 | 0.91 (0.73-1.13), 0.380 | 0.95 (0.78-1.16), 0.610 |
| Norepinephrine | 5.55 (4.26-7.24), <0.001 | 4.19 (3.48-5.04), <0.001 | 3.32 (2.87-3.85), <0.001 | 2.96 (2.58-3.39), <0.001 |
| Dopamine | 3.77 (2.93-4.85), <0.001 | 3.32 (2.71-4.06), <0.001 | 2.96 (2.49-3.56), <0.001 | 2.84 (2.39-3.37), <0.001 |
| Epinephrine | 2.25 (1.74-2.90), <0.001 | 1.79 (1.46-2.20), <0.001 | 1.44 (1.20-1.73), <0.001 | 1.34 (1.12-1.60), 0.001 |
| SOFA | 1.21(1.18-1.24), <0.001 | 1.20 (1.18-1.22), <0.001 | 1.17 (1.15-1.19), <0.001 | 1.16 (1.14-1.18), <0.001 |
| SIRS | 1.59 (1.38-1.83), <0.001 | 1.43 (1.29-1.58), <0.001 | 1.24 (1.14-1.34), <0.001 | 1.21 (1.12-1.31), <0.001 |
| APS III | 1.03 (1.03-1.03), <0.001 | 1.03 (1.03-1.03), <0.001 | 1.03 (1.03-1.03), <0.001 | 1.03 (1.03-1.03), <0.001 |
| WBC (K/µL) | 1.01 (1.00-1.02), <0.001 | 1.01 (1.00-1.01), <0.001 | 1.01 (1.00-1.01), 0.004 | 1.01 (1.00-1.01), 0.001 |
| Hemoglobin (g/dL) | 1.04 (1.00-1.09), 0.053 | 1.00 (0.97-1.04), 0.776 | 0.96 (0.93-0.98), 0.020 | 0.95 (0.93-0.97), <0.001 |
| Glucose (mg/dL) | 1.00 (1.00-1.00), <0.001 | 1.00 (1.00-1.00), <0.001 | 1.00 (1.00-1.00), <0.001 | 1.00 (1.00-1.00), <0.001 |
| Creatinine (mg/dL) | 1.08 (1.04-1.13), <0.001 | 1.10 (1.07-1.14), <0.001 | 1.11 (1.09-1.14), <0.001 | 1.12 (1.10-1.15), <0.001 |
| PaO_2_ (mmHg) | 1.00 (0.99-1.00), 0.005 | 0.99 (0.99-1.00), <0.001 | 1.00 (0.99-1.00), <0.001 | 1.00 (0.99-1.00), <0.001 |
| Bicarbonate (mmol/L) | 0.87 (0.85-0.89), <0.001 | 0.90 (0.88-0.91), <0.001 | 0.92 (0.90-0.93), <0.001 | 0.92 (0.91-0.94), <0.001 |
| ALT (IU/L) | 1.00 (1.00-1.00), <0.001 | 1.00 (1.00-1.00), <0.001 | 1.00 (1.00-1.00), <0.001 | 1.00 (1.00-1.00), <0.001 |
| Heart rate (bpm) | 1.01 (1.01-1.02), <0.001 | 1.01 (1.01-1.02), <0.001 | 1.01 (1.01-1.02), <0.001 | 1.01 (1.01-1.02), <0.001 |
| SBP (mmHg) | 0.99 (0.98-0.99), <0.001 | 0.99 (0.99-1.00), <0.001 | 0.99 (0.99-1.00), 0.007 | 1.00 (0.99-1.00), 0.038 |
| DBP (mmHg) | 1.00 (0.99-1.00), 0.264 | 1.00 (0.99-1.00), 0.690 | 1.00 (0.99-1.00), 0.846 | 1.00 (0.99-1.00), 0.844 |
| MBP (mmHg) | 0.99 (0.98-1.00), 0.003 | 0.99 (0.99-1.00), 0.002 | 1.00 (0.99-1.00), 0.052 | 1.00 (0.99-1.00), 0.037 |

MI: myocardial infarction; SOFA: sequential organ failure assessment; SIRS: systemic inflammatory response; APS III: Acute Physiology Score; WBC: white blood cell; PaO_2_: arterial oxygen partial pressures; ALT: alanine transaminase; SBP: systolic blood pressure, DBP: diastolic blood pressure, MBP: mean blood pressure,
